# Occurrence, Risk Factors, Prognosis and Prevention of Swimming-Induced Pulmonary Oedema: a Systematic Review

**DOI:** 10.1186/s40798-018-0158-8

**Published:** 2018-09-20

**Authors:** Sarah Spencer, John Dickinson, Lindsay Forbes

**Affiliations:** 1 Centre for Health Services Studies, University of Kent, Canterbury, Kent CT2 7NF UK; 2 School of Sport and Exercise Sciences, University of Kent, Chatham Maritime, ME4 4AT UK

**Keywords:** Pulmonary oedema, Immersion, Swimming, Water sports, Breathing

## Abstract

**Background:**

Swimming-induced pulmonary oedema (SIPE) can affect people with no underlying health problems, but may be life threatening and is poorly understood. The aim of this systematic review was to synthesise the evidence on SIPE incidence, prevalence, risk factors, short- and long-term outcomes, recurrence and effectiveness of interventions to prevent recurrences.

**Methods:**

We carried out a literature search using bibliographic databases and reference lists. Risk of bias was assessed by adapting existing quality assessment tools including those developed by the National Heart Lung and Blood Institute.

**Results:**

Nine studies met the inclusion criteria. Quantitative synthesis was not possible because of study heterogeneity. Five studies, which differed from each other in case definition, swimming environment, population characteristics and denominators, reported an incidence of 0.01% of UK triathlons raced over 5 years in unspecified swimming environments (one study, not fully reported, of men and women of unspecified age); 0.5% of river races swum over 3 days in Sweden (one study, of men and women up to the age of 70); and 1.8–26.7% of time trials in the sea around Israel (three studies of male teenage military trainees). One study reported that 1.4% of triathletes in the USA had experienced SIPE. One study found that hypertension, female sex, fish oil use, long course distance and another lower initial lung volumes and flows were risk factors for SIPE. A third study reported that higher mean pulmonary artery pressures and pulmonary artery wedge pressures, and lower tidal volumes were associated with SIPE. Three studies suggested that SIPE symptoms usually resolve within 24 h, although a restrictive deficit in lung function persisted for a week in one small study. We found no studies that reported deaths from SIPE. The single small study of longer-term outcomes reported no difference between affected and unaffected swimmers. Two studies suggested that around 30% of people report recurrences of SIPE. Two very small uncontrolled studies of the effect of sildenafil for recurrence prevention were inconclusive.

**Conclusions:**

SIPE may be an important public health problem affecting the growing number of recreational open water swimmers. Further research should clarify the frequency of SIPE among recreational open water swimmers, confirm reported risk factors and explore others, explore long-term consequences and test interventions to prevent recurrences.

**Electronic supplementary material:**

The online version of this article (10.1186/s40798-018-0158-8) contains supplementary material, which is available to authorized users.

## Key Points

- SIPE occurs when fluid accumulates in the lungs in the absence of water aspiration during swimming, causing acute shortness of breath and a cough productive of blood-tinged sputum.
- SIPE is likely to be a significant public health problem for open water endurance swimmers and current estimates of incidence and prevalence may be underestimates.
- High blood pressure, female sex, fish oil use, long course distance and smaller lung capacity and flows may increase risk of SIPE, although these findings need to be replicated in other studies.
- Evidence on SIPE prognosis and prevention is very limited, although it seems that most people recover quickly if removed from the water, and recurrence is common.

## Background

Open water swimming is a popular sport, with 842,500 participants in England in 2016 [1]. However, there is growing evidence that it is associated with a condition known as swimming-induced pulmonary oedema (SIPE). SIPE is a type of immersion pulmonary oedema (IPE) that occurs when fluid accumulates in the lungs in the absence of water aspiration during surface or underwater swimming, causing acute shortness of breath and a cough productive of blood-tinged sputum [2]. IPE may affect people with no underlying health problems. It is thought that in most cases symptoms subside once the individual leaves the water; however, IPE is potentially life threatening whilst a swimmer remains immersed and some IPE deaths in divers have been documented [3]. The risk of death is unknown due to difficulties differentiating between IPE and drowning for other reasons [4] and the number of cases requiring hospital treatment is unclear due to the absence of an International Classification for Diseases (ICD) diagnosis code.

IPE was first documented in scuba divers in 1981 [5]; however, there is still a lack of understanding of its occurrence, risk factors, prognosis and preventative treatments, especially in open water swimmers. IPE occurs when immersion in water creates an extrinsic pressure on the body which can force blood away from the extremities into the chest. This causes an increase in pressure gradients across pulmonary capillaries which can eventually cause a leakage of fluid into the air spaces in the lung. There is some debate around whether the different forms of IPE that occur in swimmers, scuba divers and breath-hold divers should be considered separately. Davis [6] argued that because the pathophysiological processes are the same whatever the circumstances, they should be all be termed *immersion pulmonary oedema*. However, Edmonds [7] believed the three should be separated due to the “considerable differences in the epidemiology, aquatic behaviour and physical stressors in each of these three groups”. He argued that the circumstances of an elite swimmer/triathlete swimming at the surface and breathing air are very different from a heavily equipped unfit elderly scuba diver under high hyperbaric pressures with the potential exposure to gas toxicity. Breath-hold divers are uniquely affected by a high ambient pressure on the lungs which can cause damage known as pulmonary barotrauma or lung squeeze [8]. For these reasons, we decided to restrict this review to studies of SIPE in surface swimmers.

The aim of this systematic review was to synthesise the epidemiological evidence about SIPE in open water swimmers to inform advice on risk of SIPE, implications of the diagnosis and prevention of episodes and recurrences. The following research questions were addressed:

1. What is the incidence of SIPE?
2. What is the prevalence of having experienced SIPE?
3. What are the risk factors for SIPE?
4. What are the short-term outcomes of SIPE, i.e. hospitalisation, death, recovery?
5. What is the recurrence rate of SIPE?
6. What are the long-term health sequelae of an episode of SIPE?
7. What is the evidence of the effectiveness of interventions for preventing a recurrence of SIPE?

## Methods

### Search Strategy

Peer-reviewed publications and conference abstracts were identified through online searches of the MEDLINE, EMBASE and HMIC databases using Ovid online up to April 2018 (see Additional file 1 for search strategy). Additional relevant studies were sought through The Rubicon Foundation,^1^ Mendeley, EThOS and reference lists of review papers.

### Study Selection Criteria

We included original articles and conference abstracts (where there was no full paper reporting the same data) studying human subjects that provided data to answer one or more of the research questions. Conference abstracts were included due to the small number of relevant full text articles. Initially, all abstracts that mentioned IPE were sought. These were then screened to remove articles that were not relevant. For the remaining studies, full text articles, where available, were sought. We then assessed these articles and abstracts according to the inclusion criteria shown in Additional file 2. The process was carried out independently by two reviewers.

### Data Extraction and Assessment of Study Quality

Data were extracted by both reviewers independently, using data extraction forms shown in Additional file 3. Risk of bias was assessed by adapting quality assessment tools developed by the National Heart Lung and Blood Institute [9], Hoy et al. [10] and Downs and Black [11] (see Additional file 4). Any disagreements were resolved through discussions and further reviews of studies.

## Results

**Fig. 1:**
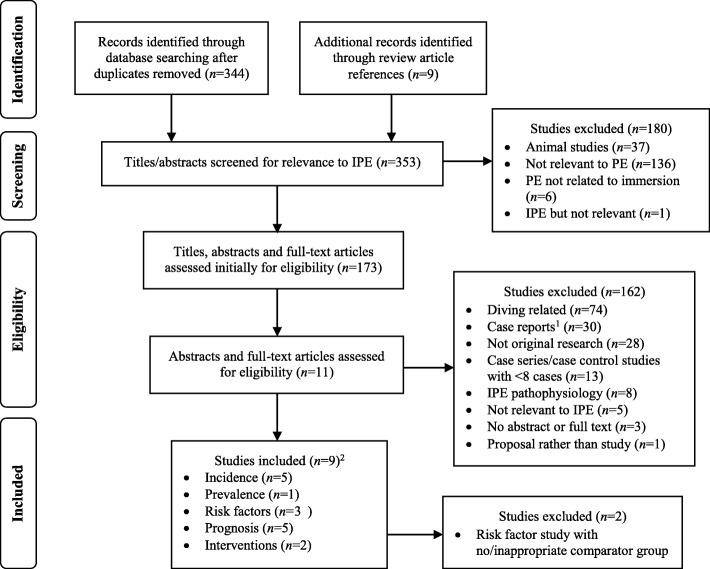
PRISMA flow diagram. *PRISMA* preferred reporting items for systematic reviews and meta-analyses; *PE* pulmonary edema; *IPE* immersion pulmonary oedema. ^1^ One case report was included in the interventions section due to a lack of relevant studies. ^2^ Numbers do not add up to total due to studies that addressed more than one research question

The search strategy identified a total of 353 titles and abstracts after duplicates were removed, of which 173 were relevant. Initial inclusion criteria were applied to these records, which included 138 full-text articles and 35 abstracts/titles, which resulted in the exclusion of 162 studies. Of the remaining 11 studies, 9 met the inclusion criteria for individual research questions (see Fig. 1).

### What Is the Incidence of SIPE?

#### Description of Studies

Five prospective incidence studies met the inclusion criteria; three of male Israeli military trainees aged 18–19 carrying out 2.4–3.6 km swimming time trials in the open-sea [12–14], one of competitors of 1–3 km swimming races in a Swedish river [15] and one of triathletes competing in UK triathlons with a 0.4–1.5 km swim [16] (see Table 1). The military trainee swimming trial studies took place over a period of 3 years, 2 months and 1 day respectively [12–14]. Braman Eriksson et al. [15] carried out their study over 3 days at the Vansbrosimningen 2016 swimming event. Smith et al. [16] studied the competitors of 11 UK triathlon races that took place over 5 years. Sample sizes of included studies ranged from 30 [14] to over 68,000 [15]. Two additional studies of divers were excluded as they did not meet the inclusion criteria; one of rebreather^2^ incidents in French military divers [17] and one of Basic Underwater Demolition/SEAL trainees at US dive training facilities [18].

**Table 1:** Incidence of SIPE

References	Study design	Subjects	Sample size and description	Type of exposure	Case definition	Case ascertainment method	Incidence reported (*n* = SIPE cases)	Critical evaluation (see Additional file 4 for more detail)
Smith et al. [16]	Prospective incidence study	Triathletes	68,557 competitors in 11 triathlon races in the UK between 2011 and 2016, distances of 400 m, 750 m and 1500 m	Not reported	Absence of water aspiration, acute onset of dyspnoea, cough and/or frothy sputum, with evidence of pulmonary oedema on physical examination	Medical records of competitors presenting to medical team	0.01% (*n* = 5) of triathlons raced	Conference abstract so limited detail. Only included competitors that sought medical assistance. No information on demographics. Unclear if any cases were recurrences in same individual
Braman Eriksson et al. [15]	Prospective incidence study	Outdoor swimmers (elite and amateur)	13,878 swimmers (6317 males, 7561 females) aged 12–70 competing in Swedish river races over 3 days in July 2016, distances of 1–3 km	Moderately cold freshwater (17 °C), unknown number of swimmers wore wetsuits	No standard definition. Examining physicians identified cases without a formal case definition.	Clinical examination of competitors presenting to medical team	Approx. 0.5% (*n* = 69) of races swum	Patient symptoms and clinical findings were not recorded. No information on competitors that did not seek medical attention
Adir et al. [12]	Prospective incidence study	Military trainees (Israeli Navy)	Unknown number of males aged 18–19 in swimming trials of 2.4–3.6 km distance (average of 30–45 min duration) in 1998–2001	Open sea of varying temperatures (19.6 °C ± 3.2), no wetsuits, supine semi-reclining position with fins	Severe shortness of breath and coughing during or after swimming in the absence of sea aspiration, and evidence of PE found on medical examination	Interview and clinical examination of swimmers presenting to medical team	1.8% (*n* = 70) of swimming trials performed	Unknown total number of swimmers and time trials. Unclear number of new cases versus recurrences
Shupak et al. [13]	Prospective incidence study	Military trainees (Israeli Navy)	35 males aged 18–19 performing 5 swimming trials (2.4–3.6 km) over 2 months (mid-Jan to mid-Mar). Trials were ≥ 1 week apart	Moderately cold open sea, (16–18 °C), diving jackets, supine position with fins	When, in the absence of prior seawater aspiration, the swimmer reported shortness of breath accompanied by coughing	Post-swim questionnaire completed by all trainees	16.6% of 175 swimming trials: 8 severe cases (4.6%), 21 mild cases (12%) 60% of swimmers (*n* = 21) had 29 episodes of SIPE	Sample size small. Study only lasted 2 months
Weiler-Ravell et al. [14]	Prospective incidence study	Military trainees (Israeli Navy)	30 males aged 18–19 performing a single 2.4 km swimming trial	Warm open sea (23 °C), no wetsuit, supine with fins, over- hydration (trainees drank approx. 5 l of water prior to swimming)	Dyspnoea and haemoptysis	Clinical examination of trainees presenting to medical team	26.7% (*n* = 8) of swimmers in one trial	Only one swimming trial. No clear case definition

*IPE* immersion pulmonary oedema, *PE* pulmonary oedema

#### Definition of SIPE

The studies used varying case definitions for SIPE apart from Braman Eriksson et al. [15] who relied on examining physicians to identify cases without a formal case definition (M Hardstedt 2018, personal communication, 13 March). These definitions of SIPE are described in Table 1. In all studies, cases of SIPE were identified from swimmers that sought medical attention, apart from Shupak et al. [13] who required all trainees, symptomatic or not, to complete post-swim questionnaires. They categorised cases into mild cases where the swimmer was able to complete the swim and severe cases where the swim had to be stopped. Different methods were used for calculating the rate of incidence. For example, some authors used the total number of swimmers as the denominator [13, 14], whereas others used the total number of swims [12, 15, 16].

#### Quality of Studies

Three studies had a clearly specified and defined population of military trainees [12–14]; the remaining two contained no demographic information on the at risk population except on identified cases [15, 16]. Inclusion and exclusion criteria were not documented in any of the studies. SIPE case definitions were mostly clearly described, although they varied considerably and the denominator used to calculate incidence varied making the results difficult to compare reliably (see “Search Strategy” section); in none of the studies were the cases censored in the subsequent analysis (in other words, cases could be counted more than once). As mentioned earlier, Shupak et al. [13] was the only study that collected data from *all* participants rather than just those that sought medical attention, making it possible to identify milder cases of SIPE. For full results of the risk of bias assessment, please see Additional file 4.

#### Findings

Quantitative synthesis was not possible due to the heterogeneous nature of the studies, in particular, the military trainees in the three studies in Israel were young men engaged in rigorous training programmes, whereas the UK and Swedish studies included recreational swimmers of all ages and both sexes. Incidence figures for SIPE varied from 0.01% of triathlons raced in the UK study [16], to 0.5% of outdoor swimming races that were swum in the Swedish study [15], to 1.8%, 16.6% and 26.7% of time trials performed by Israeli military trainees [12–14] respectively. Of the 29 cases of SIPE recorded by Shupak et al. [13], 8 were categorised as severe cases and 21 as mild, which is equivalent to 4.6% and 12.0% of swimming trials performed respectively.

### What Is the Prevalence of Having Experienced SIPE?

#### Description of Studies

Four cross-sectional surveys were identified; however, only one was of surface swimmers. This was a large survey of triathletes in the USA (Table 2) [19]. The remaining studies were of scuba divers [20] and breath-hold divers [21, 22], so they were excluded from the review. Miller et al. [19] distributed a swim-related breathing problems survey to 140,000 USA Triathlon members and received 1400 valid responses. The case definition was limited to a cough productive of pink frothy or blood-tinged secretions during swimming. Information on the type of exposure was collected, such as open water versus pool environment, use of a wetsuit, course length and climate.

**Table 2:** Prevalence of having experienced SIPE

Reference	Study design	Subjects	Sampling frame	Sample size	Response rate	Type of exposure	Case definition	Case ascertainment method	Results (*n* = SIPE cases)	Quality of evidence (see Additional file 4 for more detail)
Miller et al. [19]	Cross-sectional survey	Triathletes	140,000 members of USA Triathlon	1400 after 23 exclusions due to age < 20 or incomplete responses	1.3%	Open water/pool, wetsuit/no wetsuit, long/short course, hot/cold climate	Cough productive of pink frothy or blood-tinged secretions	Questionnaire to all participants	1.4% (*n* = 20)	Good although very low response rate and use of non-validated self-reported questionnaire

*SIPE* swimming-induced pulmonary oedema

#### Quality of Studies

Miller et al. [19] carried out a large study with a strong design, providing a detailed description of the sampling frame and methodology. The demographics of the sample appeared representative of USA Triathlon members. The analysis was robust and used appropriate numerators and denominators. However, the response rate was very low at 1.3% and data were collected through the use of a non-validated self-completed questionnaire. For full results of the risk of bias assessment, please see Additional file 4.

#### Findings

Miller et al. [19] reported a prevalence of symptoms consistent with SIPE of 1.4% of triathletes.

### What Are the Risk Factors for SIPE?

#### Description of Studies

Three studies met the inclusion criteria; a case-control study of triathletes in the USA [19], a cohort study of military trainees in Israel [13] and a small clinical trial involving 10 SIPE susceptible individuals [23] (see Table 3). An additional six studies that addressed risk factors were identified but were excluded due to not meeting the inclusion criteria, i.e. studies of scuba divers [5, 24], very small sample sizes [5], the lack of a control or comparator group [3, 12, 15] or the use of an inappropriate comparator group^3^ [25]. Miller et al. [19] used the responses from their large survey of USA Triathlon members aged ≥ 20 (85% were aged ≥ 30) augmented by 11 cases identified through slowtwitch.com (an online triathlon discussion forum), to reach a sample size of 1411 cases. Shupak et al. [13] studied a cohort of 35 military trainees (males aged 18–19) carrying out swimming time trials over a 2-month period. Moon et al. [23] studied 30 healthy subjects, of whom 10 had experienced ≥ 1 episode of SIPE during diving (2 subjects), racing or training for a triathlon (5 subjects), both (2 subjects) or after falling from a windsurfer (1 subject). Study participants carried out moderate cycle ergometer exercise for 6 to 7 min whilst submerged in water of 20 °C. All of the SIPE subjects then repeated the exercise following an oral dose of 50 mg sildenafil. Measurements of mean arterial pressure (MAP), mean pulmonary artery pressure (MPAP) and pulmonary artery wedge pressure (PAWP) were taken before immersion, during immersed exercise and shortly afterward. The types of exposure identified by Miller et al. [19] and Moon et al. [23] were very varied in terms of personal and environmental factors, whereas the participants in Shupak et al. [13] were all swimming in moderately cold open sea (16–18 °C), wearing diving jackets and fins and swimming in the supine position. Case definitions in Miller et al. [19] and Shupak et al. [13] both included a cough; however, Miller et al. [19] incorporated pink frothy or blood-tinged secretions, whereas Shupak et al. [13] specified shortness of breath and lack of aspiration of seawater. Moon et al. [23] did not report a case definition for SIPE.

**Table 3:** Risk factors associated with SIPE

References	Study design	Subjects	Sample size and description	Type of exposure	Case definition	Case ascertainment method	Exposures investigated		Findings	Quality of evidence (see Additional file 4 for more detail)
							Personal characteristics	Environmental factors		
Miller et al. [19]	Case- control	Triathletes	1400 members of USA Triathlon plus additional 11 cases (31 cases, 1380 controls)	Varying	Cough productive of pink frothy or blood-tinged secretions	Survey of USA Triathlon members plus 11 cases from slowtwitch.com	Age, sex, hypertension, diabetes, use of multivitamins, vitamin C, vitamin E, fish oil, flax oil, swimming skill, warm up, pre-swim hydration	Wetsuit use, climate trained in, open water/pool, course distance	Significant risk factors were hypertension, female sex, fish oil use and long course distance.	Self-reported non-validated tool to detect SIPE cases. Limited statistical power due to relatively small sample size. Unclear when health conditions were diagnosed and if medication being taken
Shupak et al. [13]	Prospective incidence study	Military trainees (Israeli Navy)	35 males aged 18–19 performing 5 swimming trials (2.4–3.6 km) over 2 months (mid-Jan to mid-Mar). Trials were ≥ 1 week apart	Moderately cold open sea, (16–18 °C), diving jackets, supine position with fins	When, in the absence of prior seawater aspiration, the swimmer reported shortness of breath accompanied by coughing	Post-swim questionnaire completed by all trainees	Lung function, level of exertion	None investigated	Lung volume and mid-expiratory flow measured 12 months earlier was significantly lower in SIPE susceptible group compared to asymptomatic group. No correlation between level of exertion and occurrence of SIPE	Self-reported non-validated tool to detect SIPE cases. Long period of time between screening and field study measurements. Limited statistical power due to relatively small sample size
Moon et al. [23]	Clinical trial	Triathletes, divers and one windsurfer	30 participants: 22 males, 8 females (10 cases, 20 controls)	Varying	No case definition provided	Not reported	Haemodynamics and gas exchange measurements	None investigated	SIPE group had significantly higher MPAP and PAWP, and lower tidal volume during immersed exercise	Cases had a higher proportion of females and may have been physically fitter. Inconsistency in the way pre-exercise measurements were taken between cases and controls

*SIPE* swimming-induced pulmonary oedema, *MPAP* mean pulmonary artery pressure, *PAWP* pulmonary artery wedge pressure

#### Quality of Studies

Miller et al. [19] reported good representation in terms of age and sex of participants and studied a wide range of risk factors. Shupak et al. [12] studied only two types of risk factor: pulmonary function and self-reported level of exertion, and Moon et al. [23] focussed only on haemodynamics and gas exchange measurements. A weakness of Miller et al. [19] and Shupak et al. [13] was the use of a self-reported non-validated questionnaire to detect cases of SIPE. Miller et al. [19] included questions on health conditions such as hypertension and diabetes in their questionnaire; however, it is not clear when diagnosis took place, i.e. before or after SIPE occurred, or if any medications were being taken. There is some evidence that self-reports may underestimate the prevalence of hypertension [26], although some studies have shown them to be a valid measure [27, 28]. Another shared weakness of Miller et al. [19] and Shupak et al. [12] is the lack of statistical power needed to detect significant relationships and differences between groups, due to relatively small sample sizes used. For example, Miller et al. [19] reported odds ratios of > 2 for nine risk factors; however, *p* values were low enough in only four of them to demonstrate statistical significance. Moon et al. [23] acknowledged that the SIPE group contained a much higher proportion of females than the control group and may have been physically fitter. There were also differences in the exclusion criteria used for selecting cases and controls. Additional file 4 shows full results of the quality assessment.

#### Findings

The populations studied by Miller et al. [19] and Moon et al. [23] were much more heterogeneous compared to Shupak et al. [13], i.e. male and females of varying ages versus young 18–19-year-old healthy male military recruits on rigorous training programmes. Therefore, we did not attempt quantitative synthesis. Miller et al. [19] showed the following risk factors to be associated with developing SIPE: hypertension, female sex, fish oil use and long course distance (i.e. ≥ half-ironman distance: 1.9 km swim, 90 km bike ride and 21.1 km run). Shupak et al. [13] found that lung volumes and mid-expiratory flow measured 12 months earlier were significantly lower in those who had experienced SIPE compared to the asymptomatic group. Shupak et al. [13] also reported no correlations between level of exertion and occurrence of SIPE. The results of Moon et al. [23] showed that the SIPE group had significantly higher MPAP and PAWP than non-SIPE group during immersed exercise, when accounting for differences in cardiac output. Tidal volume was also significantly lower in the SIPE group.

### Prognosis

The following research questions regarding prognosis were addressed:

1. What are the short-term outcomes of SIPE?
2. What is the recurrence rate of SIPE?
3. What are the long-term health sequelae of an episode of SIPE?

We considered short-term outcomes to be those that occurred < 30 days after an episode of SIPE, such as recovery time, hospitalisation and death. Long-term health sequelae were considered to be those which occurred ≥ 30 days following an episode.

#### Description of Studies

Five studies met the inclusion criteria; three of Israeli military trainees [12–14] (described in “What is the incidence of SIPE?” section), one of US Navy Special Warfare personnel [29] and one of outdoor swimmers in Sweden [15] (described in “What is the incidence of SIPE?” section). Ludwig et al. [29] studied a group of 20 males aged 19–36, 11 of whom had recovered from an episode of SIPE 4–14 weeks before the study commenced. Case definitions are described in Table 4. Ludwig et al. [29] had much more rigorous requirements for a diagnosis to be made compared to the other studies (see Table 4). In all studies, SIPE cases were ascertained through swimmers receiving medical attention, apart from Shupak et al. [13] who required all trainees to complete post-swim questionnaires.

**Table 4:** Prognosis of SIPE

References	Subjects	Sample size and description	Period of follow-up	Type of exposure	Case definition	Case ascertainment method	Short-term outcomes, i.e. hospitalisations and recovery	Recurrence	Long-term health sequelae	Quality of evidence (see Additional file 4 for more detail)
Adir et al. [12]	Military trainees (Israeli Navy)	Unknown number of males aged 18–19 in swimming trials of 2.4–3.6 km in 1998–2001 (70 cases)	3 years but variable	Open sea of varying temperatures (19.6 °C ± 3.2), no wetsuits, supine semi-reclining position with fins	Severe shortness of breath and coughing during or after swimming in the absence of sea aspiration, and evidence of PE found on medical examination	Interview and clinical examination of swimmers presenting to medical team	No hospitalisations. All recovered from SIPE symptoms within 24 h. Chest radiographs 12–18 h after episode were all normal. A subsample of 37 trainees had restricted lung function that persisted for a week	22.9% of cases (16 trainees) had a recurrence during the study ≥ 3 months after first episode	Not reported	No control group. Only a subsample of 37 were followed up at 7 days
Shupak et al. [13]	Military trainees (Israeli Navy)	35 males (21 cases, 14 comparators) aged 18–19 performing 5 swimming trials of 2.4–3.6 km over 2 months. Trials were ≥ 1 week apart	2 months	Moderately cold open sea, (16–18 °C), diving jackets, supine position with fins	When, in the absence of prior seawater aspiration, the swimmer reported shortness of breath accompanied by coughing	Post-swim questionnaire completed by all trainees plus physical examination: oxygen saturation, changes in pulmonary function	Not reported	31% (9 out of 29 episodes observed) were recurrences of previously observed episodes	Not reported.	No follow-up
Weiler-Ravell et al. [14]	Military trainees (Israeli Navy)	30 males (8 cases, 22 comparators) aged 18–19 performing a 2.4 km swimming trial	Until end of training programme	Warm open sea (23 °C), no wetsuit, supine position with fins, over- hydration (trainees drank approx. 5 l of water prior to swimming)	Dyspnoea and haemoptysis	Clinical examination of trainees presenting to medical team	All stayed overnight in hospital and recovered within 24 h	25% of cases (2 trainees) had a recurrence later during the training programme (unknown time period)	Not reported	Only one swimming trial. Unclear when recurrences took place
Ludwig et al. [29]	Military trainees (US Navy Special Warfare)	20 males including 11 cases aged 19–36 who had completed the first 5 weeks of a 22-week long training programme	Study began 4–14 weeks after recovery from SIPE	Not reported	(1) acute onset of dyspnoea or haemoptysis during or immediately after swimming, (2) no history of water aspiration, laryngospasm, or preceding infections, (3) hypoxemia, as defined by an oxygen saturation < 92% by pulse oximetry or an alveolar-arterial oxygen gradient of > 30 mmHg and (4) radiographic opacities consistent with an alveolar filling process and/or interstitial pulmonary oedema that resolve within 48 h	Previous clinical diagnosis of a single episode of SIPE	All recovered at least 4 weeks prior to start of study	Not reported	No significant difference in cardio-pulmonary function between cases and controls 4–14 weeks following recovery from SIPE	No SIPE symptoms observed. Small sample size. No further follow-ups
Braman Eriksson et al. [15]	Outdoor swimmers (elite and amateur)	13,878 swimmers aged 12–70 (6317 males, 7561 females) in Swedish river races over 3 consecutive days, distances of 1-3 km, Approx.69 cases of SIPE reported	N/A	Moderately cold freshwater (17 °C), unknown number of swimmers wore full wetsuits	Breathing difficulties and/or cough in the absence of clinical signs of obstruction	Clinical examination of competitors presenting to medical team	All recovered following treatment on site	31% of cases (20 swimmers) self-reported having experienced respiratory difficulties whilst swimming previously	None reported	Data only collected for those requiring treatment. Symptoms and clinical findings were not recorded for each swimmer

*SIPE* swimming-induced pulmonary oedema, *PE* pulmonary oedema, *N/A* not applicable

Only three studies reported short-term outcomes such as recovery time [12, 14, 15] and hospitalisation [14] and we found no studies that reported any deaths following SIPE. Adir et al. [12] carried out chest radiographs 12–18 h after the onset of SIPE symptoms. They also performed spirometry on a subsample of 37 trainees 6–12 h and 1 week after diagnosis of SIPE, and compared findings with baseline measurements.

Four studies provided data on recurrences, however, this was reported in different ways: Adir et al. [12] and Shupak et al. [13] reported recurrences of SIPE episodes that occurred during the study period (i.e. 3 years and 2 months respectively), whereas Weiler-Ravell et al. [14] reported recurrences that took place at unspecified times later within the same training programme, but outside the study period. Braman Eriksson et al. [15] asked participants to report their previous experience of respiratory distress whilst swimming.

Longer-term outcomes were studied only in one study: the SIPE subjects studied by Ludwig et al. [29] had all fully recovered from SIPE at least 1 month before enrolment in the study. This examined cardiopulmonary function (exercise tolerance, lung volumes and flows, pulmonary artery pressure response to hypoxaemia) at 4–14 weeks after SIPE.

#### Quality of Studies

Most of the studies were not designed as prognosis studies, and it was difficult to make comparisons between them because of differences in the outcomes described and the way in which they were reported. Apart from Braman Eriksson et al. [15], the characteristics of study participants were clearly defined in all prognosis studies. Although the sample sizes were small in the three studies of Israeli military trainees, they are likely to be representative of the military population in terms of age and sex. The representativeness of the participants of Ludwig et al. [29] and Braman Eriksson et al. [15] is unclear as no demographic information on the at risk populations were reported. For full results of our critical appraisal of these studies, please see Additional file 4.

#### Findings

The three studies that reported recovery time following SIPE found that all affected swimmers recovered from SIPE symptoms within 24 h [12, 14, 15]. Adir et al. [12] reported that chest radiographs were all normal in the first 24 h; however, the subsample of 37 trainees who had spirometry showed reduced lung volumes that persisted for a week. All eight swimmers studied by Weiler-Ravell et al. [14] that were suffering from SIPE were admitted to hospital overnight.

Reported recurrence rates in affected military trainees were 23% within 3–36 months after their first episode in one study [12], and 25% at an unspecified time during the remainder of the training programme in another [14]. The proportion of episodes of SIPE that were recurrences was 31% in two studies: in one, the previous episodes were documented [13] and in the other, the participants reported having previous experienced respiratory difficulties whilst swimming [15].

In their study of longer-term outcomes, Ludwig et al. [29] found no significant differences in cardiopulmonary function between SIPE patients and controls 4–14 weeks after the episode.

### What Is the Evidence of the Effectiveness of Interventions for Preventing a Recurrence of SIPE?

#### Description of Studies

Only two relevant studies were found; a small clinical trial [23] and a single case report [30]. Both studies examined the effect of the drug sildenafil on MAP, MPAP and PAWP. A description of the clinical trial [23] is included in “What are the risk factors for SIPE?” section. Moon et al. [23] compared outcomes before and after sildenafil in the SIPE participants. The second study was of a 46-year-old elite female ultra-triathlete who had suffered ≥ 5 episodes of SIPE [30]. The subject exercised for 6 min in 19 °C water, before and after a 50 mg oral dose of sildenafil. Measurements of MAP, MPAP and PAWP were taken at rest in dry conditions, and during immersed exercise both before and after sildenafil was administered. The woman also reported taking sildenafil before triathlons over the next 5 years and reported whether SIPE occurred.

#### Quality of Studies

Both studies were small and did not include an untreated control group with a history of SIPE. The clinical trial did not report any clinical outcomes and the intervention did not take place in “real-world” conditions. As studies of effectiveness, then, they were of low quality. A strength of the case report was that the study participant reported clinical outcomes, i.e. recurrences of SIPE over the following 5 years having taken sildenafil before triathlon events.

#### Findings

Moon et al. [23] found that before taking sildenafil, both MPAP and PAWP were higher during exercise in cold water in SIPE subjects, compared to non-SIPE subjects. After taking sildenafil, MPAP and PAWP of SIPE subjects was reduced to a level similar to that of the non-SIPE subjects. In their case report, Martina et al. [30] showed a similar effect of sildenafil in that MPAP and PAWP were lower with sildenafil than without. Following the study, the subject took sildenafil before 20 triathlons over 5 years and experienced no recurrences of SIPE symptoms.

## Discussion

### Principal Findings

We did not find conclusive evidence on the risk of developing SIPE for open water swimmers because the relevant studies differed greatly from each other in case definition, swimming environment, population characteristics and denominators [12–16, 19]. However, SIPE appears to occur in approximately 1.4% of triathletes.

It seems likely that hypertension, taking fish oil supplements and long course triathlon distance are risk factors for developing SIPE, as are non-modifiable factors such as female sex, lower lung volumes and flows, higher pulmonary artery and pulmonary artery wedge pressures, and lower tidal volumes during exercise in cold water [13, 19, 23]. However, the evidence for each risk factor comes from only one study.

The evidence suggests that SIPE symptoms normally resolve quickly following an episode [12, 14, 15]. However, we did not find any studies that reported deaths from SIPE. This may be due to difficulties differentiating between SIPE-related deaths and those caused by drowning for other reasons [4]. Three studies that reported short-term outcomes suggest that hospitalisation is not a common occurrence with 5% of cases being admitted [12, 14, 15]. Studies suggest that recurrences are common, although it is difficult to be sure of the size of the problem as the studies were in general not designed to quantify this [12–15]. One small study observed restrictive lung function defects that persisted for a week following SIPE [12]. The single small study of the long-term effects of SIPE on cardiopulmonary function only had a follow-up period of up to 14 weeks; it found no significant effects [29]. We found no studies of sequelae after 3 months. We found no conclusive evidence that sildenafil can prevent recurrences, although the studies’ results suggest that it would be appropriate to investigate this drug further for this indication [23, 30].

### Strengths and Weaknesses of the Review

To our knowledge, this is the first systematic review to gather together all the available epidemiological evidence on SIPE occurrence, risk factors, prognosis and interventions to prevent recurrences. However, our research was limited by the small number of well-designed studies that have been published. Our search strategy and inclusion criteria were broad to avoid omitting any potentially relevant publications. Our decision to exclude studies of IPE in scuba divers and breath-hold divers as well as single case reports^4^ and smaller case series/case control studies may have created a selection bias in favour of studies of young military trainees who are not representative of the general population of surface swimmers and triathletes. The exclusion of risk factor studies without an asymptomatic comparator group meant that very few risk factor studies met the inclusion criteria.

### Strengths and Weaknesses of the Available Evidence

Five studies reported greatly varying figures for the incidence of SIPE. This variation may be explained by differences between the studies in swimming environment (e.g. fresh or sea water; water temperature; nature of competition—recreational or military, for example), study participant characteristics, study length and duration of follow up, case definition and ascertainment methods, time to follow-up and methods of analysis. None of the incidence studies censored participants who had developed SIPE. Incidence rates were reported in different ways depending on whether the denominator was the number of *swims* or *swimmers*. Shupak et al. [13] distinguished between severe and mild cases of SIPE by collecting data on *all* participants, rather than just those that were seen by a clinician. This suggests that the other incidence figures reported [12, 14–16] may be underestimates. The prevalence study of USA Triathlon members [19], while fairly representative, was limited by the use of a self-completed non-validated questionnaire, a narrow case definition and a very low response rate.

Two of the risk factor studies were limited by the use of self-reported non-validated questionnaires to detect cases of SIPE and may have lacked statistical power due to the small number of SIPE cases included [13, 19]. This low statistical power meant that Miller et al. [19] were not able to demonstrate statistical significance for risk factors where odds ratios were > 2, such as age 50–59, taking vitamin C and pre-swim hydration of more than 1 l. It is also unclear if the health conditions reported by Miller et al. [19] were diagnosed before or after SIPE, or if individuals had taken medication. The third risk factor study was a small clinical trial where participants were not randomly selected and characteristics of cases and controls differed [23].

Most of the prognosis studies were not designed as such and were heterogeneous, using different case definitions, outcomes and follow-up periods [12–15, 29]. The representativeness of participants is also unclear. Our findings in relation to early recovery correspond with those of a recent summary of published case reports in which symptoms resolved within 48 h in 31 out of 38 reports (the symptoms persisting after 48 h in 7 non-resolved cases were not described) [31]. Adir et al. [12] suggested their findings of restrictive lung function deficit that persisted for a week following SIPE may indicate persistent pulmonary oedema and damage to capillaries; however, no symptoms and/or spirometric outcomes after this period were reported by the authors, so it is unclear how long the lung function deficit lasted or whether the participants had any clinical problems.

We found no studies reporting deaths of swimmers resulting from SIPE. Peacher et al. [3], in a review of published case reports, identified six published reports of deaths from IPE, but only in scuba divers. Deaths from SIPE in surface swimmers may be underestimated, especially those occurring during unsupervised training sessions in open water, which may be attributed simply to drowning. There is no separate ICD-10 code to identify death from SIPE. Harris et al. [32] studied 2971 USA Triathlon events from January 2006 to September 2008 and discovered 13 swim-related deaths that occurred during 13 triathlons; drowning was declared the cause of death for each one. In a study of 135 deaths and cardiac arrests in US triathlon participants between 1985 and 2016, Harris et al. [33] discovered 85 deaths that occurred during the swim phase. This accounted for 70% of all triathlon deaths; however, the individual causes of death were not reported for these particular cases.

Information on the long-term effects of SIPE is lacking. The single, very small, relevant study only studied patients up to 14 weeks after recovery from SIPE and did not ask the patients about their symptoms, focusing only on measures of cardiopulmonary function [29]. The findings of a study of scuba divers showed a similar result; 6 months after admission for IPE, respiratory function, cardiac echography and exercise tolerance tests were all normal [34]. However, this study also did not report symptoms, which may be present despite undetectable changes in investigations.

The studies suggest that recurrence of SIPE is common, although the different study designs used make it difficult to assess the accuracy and precision of the findings.

Two intervention studies suggested that sildenafil may be useful as a treatment to help reduce the risk of recurrence of SIPE in susceptible individuals, but the evidence is inconclusive. Neither was controlled by including an untreated control group with a history of SIPE, nor reported any clinical outcomes.

### Risk Factor Mechanisms

There has been much discussion on the mechanisms which lead to an increased risk of SIPE. The association between hypertension and IPE has been widely considered [3, 24, 35]. Hypertension can lead to diastolic dysfunction which is thought to increase the pressure in the pulmonary capillaries resulting in fluid leaking into the alveoli [2]. Wilmshurst et al. [24] found that blood pressure in a group of 11 SIPE susceptible divers became significantly higher than the control group following exposure to cold and/or raised partial pressure of oxygen. Peacher et al. [3] carried out a literature review of 292 cases of pulmonary oedema in swimmers and divers, as well as a case series study (*n* = 36). They found that 16% of the recreational swimmers and divers were reported to have hypertension, as were 17% of cases in their case series. Gempp et al. [35] found that 23% of the 73 scuba divers treated for IPE at a French hyperbaric facility suffered from hypertension. However, these hypertension prevalence figures for IPE cases reported by Peacher et al. [3] and Gempp et al. [35] are lower than World Health Organisation region crude estimates (Europe: 28.1%, Americas: 18.8%, Eastern Mediterranean: 22.0%) [36], so do not show a clear link between hypertension and IPE.

The connection between SIPE and fish oil consumption is believed to be caused by its anti-platelet and vasodilatory effect which can make it easier for fluid to pass into the pulmonary capillaries [19]. The use of the anti-platelet medication aspirin in breath-hold divers has also been linked to three cases of SIPE in a small case series [37]. Miller et al. [19] suggested that the relationship between long course distance and SIPE may be related to wetsuit use, as the authors were unable to separate the effects of these two factors. The relationship between IPE and female sex was discussed by Coulange et al. [34] who reported a statistically higher incidence of pulmonary oedema in female scuba divers. Guenette et al. [38] tested the response to exercise of endurance trained athletes and found there were disadvantages in pulmonary response to exercise of females that was probably due to lung size. Hopkins et al. [39] suggested smaller lung size might be the mechanism by which females are at a higher risk of pulmonary oedema. The findings of Shupak et al. [13] that affected military trainees had significantly smaller initial lung volumes and flows suggests that even in males, smaller lung volumes may lead to a greater risk of SIPE. In a study of a small number of SIPE and high-altitude pulmonary oedema (HAPE) subjects and controls, Carter et al. [40] found no significant difference in lung volumes; however, the SIPE/HAPE subjects *did* have a lower lung density and mass, and fewer pulmonary lymphatics. The authors hypothesised that a limited pulmonary lymphatic system was less able to reabsorb excess fluid that leaked into the interstitial and alveolar space, leading to a greater risk of pulmonary oedema.

### Recommendations for Future Research

Future studies of the frequency of SIPE should focus on those with milder symptoms in order to reflect the true burden of this condition. Larger studies that can replicate the findings on hypertension, fish oil consumption, long course distance, lower lung volumes and flows, higher pulmonary artery pressures and pulmonary artery wedge pressures and lower tidal volumes during exercise in cold water, as risk factors for SIPE, are needed to improve our understanding of SIPE. Future research should explore other proposed risk factors such as wetsuit use, age, over hydration, water temperature, swimming position, exertion, presence of specific genes variants, failure to warm up and variations in stress response. Future studies must also examine the effects of SIPE on the cardiorespiratory system over the months and years after the initial or repeated episode, including studies of symptoms as well as cardiovascular and respiratory function tests. Sildenafil has showed promise as a potential prophylactic against SIPE; the drug now needs to undergo randomised controlled trials to determine its efficacy.

## Conclusions

The evidence on the occurrence of SIPE suggests that it is an important public health issue for recreational endurance open water swimmers, clinicians and event organisers, which is particularly important given the popularity of this sport in the UK and other countries. It is likely that current estimates are underestimates of the true burden of this condition. It appears that recurrence rates are high, so it is important to advise SIPE patients of this and recommend close supervision by others who can help them get out of the water if affected.

## Additional Files

Additional file 1: Search strategy using Ovid online. (DOCX 18 kb)

Additional file 2: Inclusion criteria. (DOCX 14 kb)

Additional file 3: Data extraction forms. (DOCX 17 kb)

Additional file 4: Results of assessments of risk of bias. (DOCX 23 kb)

## Abbreviations

HAPE: High-altitude pulmonary oedema
ICD: International Classification for Diseases
IPE: Immersion pulmonary oedema
MAP: Mean arterial pressure
MPAP: Mean pulmonary artery pressure
PAWP: Pulmonary artery wedge pressure
PE: Pulmonary edema
SIPE: Swimming-induced pulmonary oedema

## Footnotes

1 The Rubicon Foundation is a non-profit organisation interested in research, exploration, science and education which hosts a digital repository of diving and environmental physiology research.

2 Rebreathers absorb the carbon dioxide exhaled by a diver whilst recycling the unused oxygen content and replenishing it for the next breath.

3 Participants of the 1985 to 1995 Hawaii Ironman World Championships were deemed unrepresentative of the overall triathlete population.

4 Case reports *were* included in the section addressing interventions to prevent SIPE recurrences due to the very limited amount of relevant literature
