# Correction to: Occurrence, Risk Factors, Prognosis and Prevention of Swimming-Induced Pulmonary Oedema: a Systematic Review

**DOI:** 10.1186/s40798-019-0184-1

**Published:** 2019-03-27

**Authors:** Sarah Spencer, John Dickinson, Lindsay Forbes

**Affiliations:** 1 Centre for Health Services Studies, University of Kent, Canterbury, Kent CT2 7NF UK; 2 School of Sport and Exercise Sciences, University of Kent, Chatham Maritime, ME4 4AT UK

**Correction to: Sports Med Open**

**https://doi.org/10.1186/s40798-018-0158-8**

The original article [1] contained a minor error in Fig. 1. The corrected figure is shown below.

**Fig. 1:**
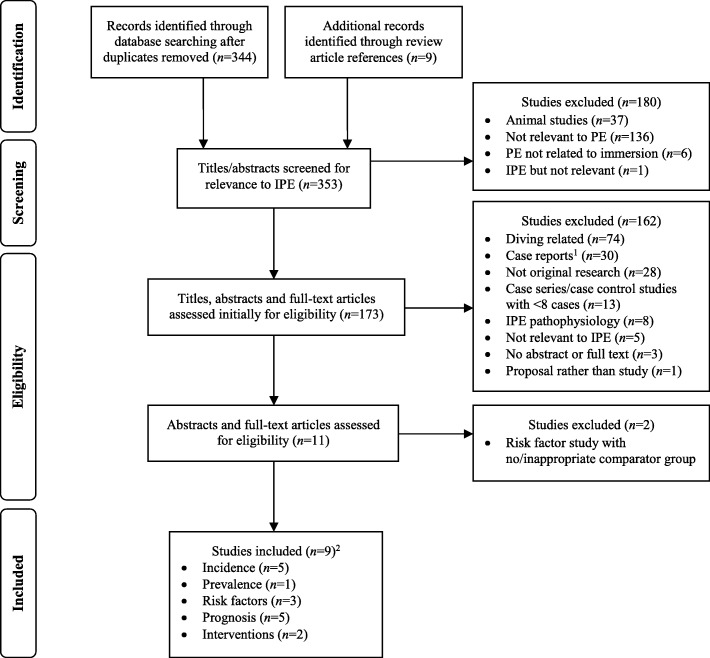
PRISMA flow diagram. PRISMA Preferred Reporting Items for Systematic Reviews and Meta-Analyses. IPE: immersion pulmonary oedema; PE: pulmonary oedema. ^1^ One case report was included in the interventions section due to a lack of relevant studies. ^2^ Numbers do not add up to total due to studies that addressed more than one research question
