# Supplementary material for: Occurrence, Risk Factors, Prognosis and Prevention of Swimming-Induced Pulmonary Oedema: a Systematic Review
Source: Sports Med Open. 2018 Sep 20;4:43. doi: 10.1186/s40798-018-0158-8 (PMC6146959; doi:10.1186/s40798-018-0158-8)
Supplement: Supplementary file 1 — Search strategy using Ovid online. (DOCX 18 kb) [file 40798_2018_158_MOESM1_ESM.docx]

**Additional files**

**Additional file 1: Search strategy using Ovid online**

| **Search number** | **Search Terms** | **Results** |
| --- | --- | --- |
| 1 | (immersion or submersion or swim* or diver or divers or diving or triath* or scuba).af.ab.dm.dv.fx.kw.mf.nm.ot.px.rx.sy.ti.tn.ui. | 166,825 |
| 2 | (pulmonary adj (oedema or edema)).af.ab.dm.dv.fx.kw.mf.nm.ot.px.rx.sy.ti.tn.ui. | 51,704 |
| 3 | 1 and 2 | 531 |
| 4 | Remove duplicates from 3 | 352 |
