# Supplementary material for: Occurrence, Risk Factors, Prognosis and Prevention of Swimming-Induced Pulmonary Oedema: a Systematic Review
Source: Sports Med Open. 2018 Sep 20;4:43. doi: 10.1186/s40798-018-0158-8 (PMC6146959; doi:10.1186/s40798-018-0158-8)
Supplement: Supplementary file 2 — Inclusion criteria. (DOCX 14 kb) [file 40798_2018_158_MOESM2_ESM.docx]

**Additional file 2: Inclusion criteria**

| **Population:** | Surface swimmers of any age swimming in pools or open-water of any temperature. Excluding scuba or breath-hold (apnoea) divers. |
| --- | --- |
| **Case definition:** | Clinical diagnosis of SIPE or symptoms suggestive of SIPE (acute onset of shortness of breath during swimming, with or without cough, with or without sputum production, in the absence of water aspiration). Care was taken to differentiate SIPE from other causes of acute breathlessness; most commonly asthma, cold shock response, left ventricular failure, pulmonary embolism and pneumonia. |
| **Study design:** | *Incidence:* Studies which identified a population of people at risk and followed them up to identify whether they developed SIPE.  *Prevalence:* Cross-sectional studies carried out at a single point in time or over a defined period of time, in which cases of SIPE were sought using a consistent case definition.  *Risk factors:* Cohort or case-control studies of people who had experienced SIPE and a comparator group of swimmers with no history of SIPE or symptoms suggestive of SIPE, where the number of cases ≥8. This number was selected due to the low likelihood of finding statistical associations with <8 cases and an effort to ensure that not all studies were excluded. Exposures included personal characteristics such as age, sex, body mass index, body fat, medical history, medication, smoking etc. or environmental factors such as water temperature, weather conditions, clothing worn e.g. wetsuit, dive jacket, swimsuit etc.  *Prognosis:* Any studies where there was a group of patients meeting the case definition and short (i.e. within 30 days) or long term outcomes were reported. Short term outcomes of interest included treatment, hospitalisation, length of time to recovery or death. Long term outcomes of interest included recurrence of SIPE and long term sequelae.  *Interventions:* Any studies of interventions for the prevention of SIPE recurrences. |
