# Supplementary material for: Occurrence, Risk Factors, Prognosis and Prevention of Swimming-Induced Pulmonary Oedema: a Systematic Review
Source: Sports Med Open. 2018 Sep 20;4:43. doi: 10.1186/s40798-018-0158-8 (PMC6146959; doi:10.1186/s40798-018-0158-8)
Supplement: Supplementary file 3 — Data extraction forms. (DOCX 17 kb) [file 40798_2018_158_MOESM3_ESM.docx]

**Additional file 3: Data extraction forms**

***Incidence***

| Study ID number |  |
| --- | --- |
| Author(s) |  |
| Institution |  |
| Title |  |
| Publisher |  |
| Abstract |  |
| Study design |  |
| Publication Type |  |
| Subjects |  |
| Period of data collection |  |
| Country |  |
| Population size |  |
| Sampling frame |  |
| Sample size |  |
| Description of study population |  |
| How sample chosen/inclusion criteria |  |
| Exclusions |  |
| How cases identified |  |
| Case definition |  |
| No. cases identified |  |
| Incidence (%) |  |
| Water temp |  |
| Depth |  |
| Open water/pool |  |
| Wetsuit use |  |
| Mean age |  |
| Health conditions |  |
| Duration of swim/dive |  |
| Findings |  |
| Quality |  |
| Include in review? |  |

***Prevalence***

| Study ID number |  |
| --- | --- |
| Author(s) |  |
| Institution |  |
| Title |  |
| Publisher |  |
| Abstract |  |
| Study design |  |
| Publication Type |  |
| Subjects |  |
| Type of activity |  |
| Period of data collection |  |
| Country |  |
| Population size |  |
| Sampling frame |  |
| Sample size |  |
| Description of study population |  |
| How sample chosen/inclusion criteria |  |
| Exclusions |  |
| How cases identified |  |
| Case definition |  |
| No. cases identified |  |
| Prevalence (%) |  |
| Water temp |  |
| Depth |  |
| Open water/pool |  |
| Wetsuit use |  |
| Mean age |  |
| Health conditions |  |
| Duration of swim/dive |  |
| Findings |  |
| Quality |  |
| Include in review? |  |

***Risk factors***

| Study ID number |  |
| --- | --- |
| Author(s) |  |
| Institution |  |
| Title |  |
| Publisher |  |
| Abstract |  |
| Study design |  |
| Description of study population |  |
| Risk factors studied |  |
| Period of data collection |  |
| Country |  |
| Population size |  |
| Sampling frame |  |
| Sample size |  |
| How sample chosen/inclusion criteria |  |
| Exclusions |  |
| How cases identified |  |
| Case definition |  |
| No. cases identified |  |
| Comparator/control? |  |
| Water temp |  |
| Open water/pool |  |
| Wetsuit use |  |
| Mean age |  |
| Health conditions |  |
| Previous respiratory distress whilst swimming |  |
| Medication/supplement use |  |
| Duration of swim/dive |  |
| Pre-swim hydration |  |
| Warm up |  |
| Other risk factors |  |
| Findings |  |
| Quality |  |
| Include in review? |  |

***Prognosis***

| Author(s) |  |
| --- | --- |
| Institution |  |
| Title |  |
| Source |  |
| Publisher |  |
| Abstract |  |
| Study design |  |
| Country |  |
| Description of participants |  |
| Activity |  |
| Number of cases |  |
| About recurrence/recovery from acute episode/ long term sequelae |  |
| Period of follow up |  |
| Completeness of follow up |  |
| Recovery from initial acute episode |  |
| How recurrence identified |  |
| Definition of recurrence |  |
| Number of recurrences <30 days |  |
| Number of recurrences 30 days + |  |
| Other sequelae |  |
| Quality |  |
| Include in review? |  |
