# Supplementary material for: Occurrence, Risk Factors, Prognosis and Prevention of Swimming-Induced Pulmonary Oedema: a Systematic Review
Source: Sports Med Open. 2018 Sep 20;4:43. doi: 10.1186/s40798-018-0158-8 (PMC6146959; doi:10.1186/s40798-018-0158-8)
Supplement: Supplementary file 4 — Results of assessments of risk of bias. (DOCX 23 kb) [file 40798_2018_158_MOESM4_ESM.docx]

**Additional file 4: Results of assessments of risk of bias**

***Incidence (adapted from NHLBI, 2014)***

| **Reference** | **Was the study population clearly specified and defined?** | **Were all the subjects selected or recruited from the same or similar populations (including the same time period)?** | **Were inclusion and exclusion criteria for being in the study pre-specified and applied uniformly to all participants?** | **Were the exposure measures (independent variables) clearly defined, valid, reliable, and implemented consistently across all study participants?** | **Were the outcome measures (dependent variables) clearly defined, valid, reliable, and implemented consistently across all study participants?** | **Was loss to follow-up after baseline 20% or less.** |
| --- | --- | --- | --- | --- | --- | --- |
| Smith et al 2017 | No - 68,557 competitors of 11 triathlons including elite and non-elite competitors. No detail on demographics other than cases of SIPE. | Yes - All subjects were triathletes although time periods for individuals were different depending on the races entered over the 5 years of the study. | No inclusion/exclusion criteria other than race entry requirements. | No - crude measure of exposure: total number of races started by competitors in 2011-2016. No detail on individual races such as number of competitors and distance. No information on the number of races started by individual competitors. | Yes - SIPE clearly defined and identified from medical records. Only applied to triathletes seeking medical attention. Unknown if triathletes had previously experienced SIPE. | No - data only collected for competitors seeking medical attention |
| Braman Eriksson et al 2017 | No - 13,878 competitors at a 3 day open-water swimming event. No detail on demographics other than cases of SIPE. | Yes – Subjects were all open-water swimmers including some multi-sport endurance athletes. Races were all within 3 days. | No inclusion/exclusion criteria other than race entry requirements | No - crude measure of exposure: total number of races started by competitors over 3 days. Races of 1000, 1500 & 3000 metres. No detail on individual races such as number of competitors and distance. No information on the number of races started by individual competitors. | No – examining physicians identified SIPE cases without a formal case definition. Records not kept for every patient. Some swimmers had a previous history of respiratory distress although unclear if this was SIPE. | No - data only collected for competitors seeking medical attention. |
| Adir et al 2004 | Yes – 70 military trainees undergoing swimming time trials between 1998 and 2001 diagnosed with SIPE. No data on trainees without SIPE symptoms. All healthy fit males aged 18-19. | Yes - All subjects were military trainees undergoing a fitness training programme within the 4 years study period. | No inclusion/exclusion criteria other than military requirements. | No – exposure i.e. number of time trials undertaken, not known. No detail on individual time trials except that distances were 2.4-3.6km and of 30-45 mins duration. | Yes - SIPE clearly defined and identified from symptoms and clinical examination. Unclear if participants had experienced SIPE prior to the study. | No - data only collected for SIPE cases |
| Shupak et al 2000 | Yes - 35 healthy fit military trainees undergoing swimming time trials over 2 months. All males aged 18-19. | Yes - All subjects were military trainees undergoing the same 2 month fitness training programme. | No inclusion/exclusion criteria other than military requirements. | Partly - exposure measured as the total number of time trials undertaken, although unclear how many each trainee swam. Distances were all 2.4-3.6km. | Yes - SIPE clearly defined and detected through a non-validated self-complete questionnaire which also enabled differentiation into severe and mild cases. Unknown if participants had experienced SIPE prior to the study. | Yes – no loss to follow-up after baseline |
| Weiler-Ravell 1995 | Yes – 30 military trainees undergoing a swimming time trial. All males aged 18-19. | Yes – All subjects were military trainees undergoing the same swimming time trial. | No inclusion/exclusion criteria other than military requirements. | Yes – exposure was the same for all swimmers: distance of 2.4km in 23°C calm open sea waring bathing suit with very high hydration levels | Yes – SIPE clearly defined and identified from symptoms observed. Unknown if participants had experienced SIPE prior to the study. | Yes – no loss to follow-up after baseline. |

***Prevalence (adapted from Hoy et al, 2012)***

| **Reference** | **Was the sample representative of the target population? (selection bias)** | **Was the likelihood of non-response bias minimal?** | **Was the study instrument that measured the parameter of interest shown to have validity and reliability? (measurement bias)** | **Was the same mode of collection used for all subjects? (measurement bias)** | **Were the numerator(s) and denominator(s) for the parameter of interest appropriate? (analysis bias)** |
| --- | --- | --- | --- | --- | --- |
| Miller et al 2010 | Yes – large sample size (n=1400) and no sig. difference between age distribution of sample and target population. Exclusions: <20 yrs, incomplete responses. | No – very low response rate of 1.3% | No - all data collected through self-completed non-validated standardised questionnaire. | Yes | Yes – numerator was number of respondents reporting history of symptoms of SIPE, denominator was all respondents |

***Risk factors (adapted from Downs and Black, 1998)***

| **Reference** | **Are the characteristics of the participants included in the study clearly defined?** | **Were the subjects who were prepared to participate representative of the entire population from which they were recruited? (selection bias)** | **Were the cases and controls/comparators recruited from the same population? (selection bias)** | **Were the main outcome measures accurate (valid and reliable)? (measurement bias)** | **Were the statistical tests used to assess main outcomes appropriate? (analysis bias)** | **Did the study have sufficient power to detect a clinically important effect where the probability value for a difference being due to chance is less than 5%?** |
| --- | --- | --- | --- | --- | --- | --- |
| Miller et al 2010 | Yes - survey respondents are clearly defined (however no information on the additional 11 cases from slowtwitch) | Yes – no sig diff between age distribution of survey sample (n=1400) and target population (representativeness of 11 cases from slowtwitch is unclear) | Mostly – Controls were survey respondents but cases were made up of both survey respondents and 11 slowtwitch contributors | No – outcome measure of SIPE was detected using non-validated self-completed questionnaire | Yes – exploration of univariate risk factors and use of multiple logistic regression to determine predictors of SIPE | Yes |
| Shupak et al 2000 | Yes - 35 healthy fit military trainees undergoing swimming time trials over 2 months. All males aged 18-19. | Yes – although the sample size is small, it is likely to be representative of the military trainee population in terms of age and gender | Yes – cases and comparators from within the cohort | Self-reported level of exertion was subjective, lung function tests used calibrated spirometer | Yes – Spearman’s rank test for correlation between exertion and occurrence of SIPE, one way ANOVA and Tukey test to compare lung function between groups | Yes |
| Moon et al 2016 | Yes – subject characteristics are reported. | Unclear – no comparisons are made between participants and the target population.  Unknown how SIPE subjects were chosen. | No – although cases and controls were similar in age, BMI and race, cases had a higher proportion of females and may have been physically fitter. | Inconsistency in the way pre-exercise measurements were taken i.e. SIPE subjects were supine while controls were sitting. | Yes - appropriate statistical tests were used to analyse the data. | Yes |

***Prognosis***

| **Reference** | **Are the characteristics of the participants included in the study clearly defined?** | **Were the subjects who were prepared to participate representative of the entire population from which they were recruited? (selection bias)** | **Were the main outcome measures accurate (valid and reliable)? (measurement bias)** | | |
| --- | --- | --- | --- | --- | --- |
|  |  |  | **Short term outcomes i.e. hospitalisations and recovery** | **Recurrence** | **Long term sequelae** |
| Adir et al 2004 | Yes – 70 military trainees undergoing swimming time trials between 1998 and 2001 who were diagnosed with SIPE. All healthy fit males aged 18-19. | Yes – although the sample size is small, it is likely to be representative of the military trainee population in terms of age and gender | Yes, resolution of SIPE symptoms within 24 hours reported. Also included tests of lung function and echocardiographic investigations. | Yes, recurrences reported within 3 years of study | Not reported |
| Shupak et al 2000 | Yes - 35 healthy fit military trainees undergoing swimming time trials over 2 months. All males aged 18-19. | Yes – although the sample size is small, it is likely to be representative of the military trainee population in terms of age and gender | Not reported | Yes, recurrences reported within the 2 months of the study | Not reported |
| Weiler-Ravell et al1995 | Yes – 30 healthy fit military trainees undergoing a swimming time trial. All males aged 18-19. | Yes – although the sample size is small, it is likely to be representative of the military trainee population in terms of age and gender | Yes, hospitalisations and resolution of SIPE symptoms within 24 hours reported | No, although recurrences reported during the remainder of the training programme, time lag is unknown. | Not reported |
| Ludwig et al 2006 | Yes – 20 healthy fit military trainees aged 19-36 who had completed the first 5 weeks of a 22 week long Basic Underwater Demolition/SEAL training programme | Unclear – no demographic information on at risk population other than they are all male | Short term outcomes were not described, although all SIPE patients had recovered at least 4 weeks before study | Not reported | Partly. Measures of cardiopulmonary function compared to non-SIPE subjects |
| Braman Eriksson et al 2017 | No - 13,878 swimmers at a 3 day open-water swimming event. No detail on demographics other than cases of SIPE. | Unclear – no demographic information on the at risk population of open-water swimmers including multi-sport endurance athletes. | Yes, reported that all symptoms resolved following treatment on-site. | Partly. Reported as number of swimmers who had experienced symptoms before (not necessarily clinically diagnosed) | Not reported |

***Interventions***

| **Reference** | **Are the characteristics of the participants included in the study clearly defined? (selection bias)** | **Were the subjects who were prepared to participate representative of the entire population from which they were recruited? (selection bias)** | **Were inclusion and exclusion criteria for being in the study pre-specified and applied uniformly to all participants? (selection bias)** | **Were the cases and controls/comparators recruited from the same population? (selection bias)** | **Were the main outcome measures accurate (valid and reliable)? (measurement bias)** |
| --- | --- | --- | --- | --- | --- |
| Moon et al 2016 | Yes – age, gender and exemptions described in detail | Unknown how SIPE subjects were chosen. | No. Body fat criterion not applicable to SIPE subjects and mild hypertension allowed in SIPE subjects if controlled by medication. | No sig diffs in age, BMI and race, but sig greater proportion of males in control group. IPE group may have been fitter than non-IPE group. | No. No untreated control group with a history of SIPE. Inconsistency in the way pre-exercise measurements were taken i.e. SIPE subjects were supine while non-SIPE subjects were sitting. No clinical outcomes reported. |
| Martina et al 2017 | Yes, but only one participant. | N/A | N/A | N/A | No. Only one case so low quality study. |
